
JOURNAL INFORMATION
==============================
NLM Title Abbreviation: ScientificWorldJournal
Journal ID: ScientificWorldJournal
NLM Journal ID: 101131163
Journal ID: TSWJ

The Scientific World Journal

ISSN: 2356-6140
EISSN: 1537-744X
Publisher: Wiley

ARTICLE INFORMATION
==============================
PMCID: PMC5917207
PMID: 16964367
DOI: 10.1100/tsw.2006.219
Article ID: 938735
Article version: 1
Subjects: Directions in Science

Guidance of Neural Crest Cell Migration: The Inhibitory Function of the Chondroitin Sulfate Proteoglycan, Versican

Dutt Shilpee 1 2
Matasci Mattia 1
Sommer Lukas 3
Zimmermann Dieter R. 1 *
1 Laboratory of Molecular Biology Department of Pathology University Hospital Zurich, Switzerland
2 Department of Laboratory Medicine Children's Hospital Harvard Medical School, Boston, MA, United States
3 Institute of Cell Biology Department of Biology Swiss Federal Institute of Technology, Zurich, Switzerland
*Dieter R. Zimmermann: dieterzi@pathol.unizh.chAcademic Editor: Martin Goette

Publication date: 2006
Electronic publication date: 2006 Sep 6
Volume: 6
First page: 1114
Last page: 1117
Received 2006 Jul 17; Revised 2006 Aug 21; Accepted 2006 Aug 31
Copyright: Copyright © 2006 Shilpee Dutt et al.
Copyright year: 2006
License: This is an open access article distributed under the Creative Commons Attribution License, which permits unrestricted use, distribution, and reproduction in any medium, provided the original work is properly cited.
License URL: https://creativecommons.org/licenses/by/3.0/

Keywords: embryogenesis, neural crest cells, migration, guidance, cell adhesion, extracellular matrix, proteoglycan, hyalectan, versican

Funding: Swiss National Science Foundation Velux Foundation National Center of Competence in Research

==============================
Directions in Science
*TheScientificWorldJOURNAL* (2006) 6, 1114–1117
ISSN 1537-744X; DOI 10.1100/tsw.2006.219

TheScientificWorldJOURNAL
www.thescientificworld.com

### Guidance of Neural Crest Cell Migration: The Inhibitory Function of the Chondroitin Sulfate Proteoglycan, Versican

Shilpee Dutt[1,2§], Mattia Matasci[1§], Lukas Sommer[3], and Dieter R. Zimmermann[1,*]

*[1]Laboratory of Molecular Biology, Department of Pathology, University Hospital Zurich, Switzerland; [2]Department of Laboratory Medicine, Children's Hospital, Harvard Medical School, Boston; [3]Institute of Cell Biology, Department of Biology, Swiss Federal Institute of Technology, Zurich, Switzerland*

**E-mail:** dieterzi@pathol.unizh.ch

*Received July 17, 2006; Revised August 21, 2006; Accepted August 31, 2006; Published September 6, 2006*

---

**KEYWORDS:** embryogenesis, neural crest cells, migration, guidance, cell adhesion, extracellular matrix, proteoglycan, hyalectan, versican

---

Neural crest cells are specialized multipotent embryonic stem cells found exclusively in vertebrates[1,2,3]. During embryonic development, these cells arise from the dorsal neural tube, undergo epithelial to mesenchymal transition, and subsequently migrate along stereotyped pathways to reach specific tissue targets, where they differentiate into a wide variety of cell types, such as glia and neurons of the peripheral nervous system, melanocytes, smooth muscle cells, craniofacial cartilage and bone tissues, or chromaffin cells of the adrenal medulla. In the trunk region, the ventrally migrating neural crest cells move through the somitic mesenchyme in a segmented pattern, presumably setting the basis for the metameric organization of sensory and sympathetic ganglia along the anterior-posterior axis later in development[4]. Early grafting experiments have shown that this highly specific migration behavior is primarily controlled by the microenvironment provided by the surrounding mesenchymal cells and their extracellular matrix. Different nonpermissive tissues forming barriers to neural crest cell movement have been identified, including the posterior sclerotome[5], the perinotochordal region[6], and transiently, the tissue underneath the dorsolateral ectoderm[7]. These boundaries contain, like the pathway tissues, migration-promoting proteins, such as fibronectin and laminins, but additionally include various cell surface and extracellular matrix components that may inhibit neural crest cell adhesion and invasion and, hence, maintain the moving cells within their trajectory. Among the candidate inhibitors are chondroitin 6-sulphate proteoglycans (CSPGs), peanut agglutinin (PNA)-binding glycoproteins[8], F-spondin[9], T-cadherin[10], secreted class 3 semaphorins[11,12], and ephrins[13,14]. Although they are selectively expressed in barrier tissues during the phase of neural crest cell migration, evidence for their direct involvement in the guiding process are only now emerging. The current findings suggest that the guidance of neural crest cell migration is not controlled by a single inhibitor, but rather depends on the concerted action of multiple factors that may jointly regulate different aspects of neural crest migration.

Our laboratory previously contributed to the identification of such candidate inhibitors by showing that most of the chondroitin-6-sulfate immunoreactivity and possibly also the PNA-binding carbohydrates are in barrier tissues of chick embryos linked to the presence of the large V0 and V1 splice-variants of the

*Corresponding author.
[§]both authors contributed equally to this review.

©2006 with author.
Published by TheScientificWorld, Ltd.; www.thescientificworld.com

CSPG versican[15]. We then demonstrated that the versican expression closely correlates with the formation of nonpermissive extracellular matrices within the caudal sclerotome, the early subectodermal tissue, and to a lesser extent, around the notochord, where another member of the hyalectan family, aggrecan, may be the predominant CSPG[16].

In extension of this study, we recently published in *The Journal of Biological Chemistry* new data that provide clear evidence for an active involvement of versican V0 and V1 in the guidance process[17]. Using stripe choice assays, we demonstrated that isolated versicans strongly inhibit the migration of Sox10/p75NTR double-positive neural crest stem cells on fibronectin by interfering with cell-substrate adhesion. We also showed that this inhibition is largely core glycoprotein dependent, as the complete removal of the glycosaminoglycan chains had only a minor effect on the inhibitory capacity.

Perris et al. showed that surface-bound aggrecan or versican alone are not supportive for neural crest cell migration[18]. Our data now expand this finding to demonstrate that intact versican isoforms V0 and V1 are not simply nonpermissive substrates, but actively override the adhesion- and migration-promoting capacity of fibronectin, which has been coated together with versicans in all of our experiments. Interestingly, we noted an inhibitory activity already at very low coating concentrations (less than 25 μg/ml), while Perris et al. reported that versican added to the cell culture medium in solution displayed, in contrast to aggrecan, no such effect even at very high concentrations of up to 3 mg/ml. This discrepancy either indicates that the large versican isoforms exert their inhibitory activity only after immobilization in a fibronectin-containing extracellular matrix, or may be linked to a partial degradation of the versican preparations used in their experiments. Similarly, this might explain why Perissinotto et al.[19] did not observe an inhibitory effect in a study implanting filters soaked in versican solutions into chick embryos, leading them to propose rather a “haptotactic-like” substrate function for versicans during the migration of neural crest cells (a notion that we could not confirm). A direct comparison of the versican concentrations effective in our assays with the amounts ultimately deposited in their experiments is difficult because of differences between coating and binding of versicans to the filter material they used for implantation (Nuclepore).

Since no cell surface receptors for versicans are currently known, the exact mechanism by which versicans V0/V1 may negatively control neural crest cells migration remains still elusive, but is likely to be directly or indirectly connected to the function of integrins and to Rho/Rac-mediated reorganization of the cytoskeleton[20]. It seems possible that versican cooperates with other known inhibitors belonging to the ephrin or the semaphorin families. Eph receptors expressed by migrating neural crest cells and ephrins present in barrier tissues are believed to participate in the induction of the repulsive response. This notion is mainly based on *in vitro* experiments with isolated neural crest cells and with chick trunk explant cultures[13]. No defects in the metameric patterning of neural crest cells migration have been observed in ephrin or Eph receptor knockout mice, however[21,22,23]. Similarly, neural crest cells avoid semaphorin 3A[11] and semaphorin 3F[12] *in vitro*. While the knockout of Sema 3A does not result in alterations of the neural crest cell migration[24], inactivation of the genes encoding either semaphorin 3F or its receptor, neuropilin-2, leads to the disruption of the segmental migration pattern through the somites of mutant mice[12]. Nevertheless, the metameric pattern of the sensory dorsal root ganglia is not perturbed, again suggesting a more complex regulatory process.

The recent studies from our and other laboratories have just begun to unravel the molecular mechanism behind the control of neural crest cell migration. At present, many important questions that could explain their specialized moving behavior *in vivo* remain open, for example: (1) What are the relative contributions of the different factors in the guidance of neural crest cells? (2) What are the pathways that transduce the inhibitory signal within the cell? (3) Are neural crest cells responding all in the same way to a single inhibitor or are discrete subpopulations reacting differentially to distinct extracellular cues? The analysis of mouse models with multiple gene knockouts generated by combining conditional and constitutive gene inactivation approaches and the application of novel time-lapse imaging techniques in explant cultures or directly *in vivo* may help to answer these questions in the future.

### ACKNOWLEDGMENT

Our work has been financed in part by grants from the Swiss National Science Foundation and the Velux-Foundation (D.R.Z.), and grants from the Swiss National Science Foundation and from the National Center of Competence in Research "Neural Plasticity and Repair" (L.S).

### REFERENCES

1. Knecht, A.K. and Bronner-Fraser, M. (2002) Induction of the neural crest: a multigene process. *Nat. Rev. Genet.* **3**, 453–461.
2. Le Douarin, N.M. (2004) The avian embryo as a model to study the development of the neural crest: a long and still ongoing story. *Mech. Dev.* **121**, 1089–1102.
3. Crane, J.F. and Trainor, P.A. (2006) Neural crest stem and progenitor cells. *Annu. Rev. Cell Dev. Biol*. **22**, 267–286.
4. Krull, C.E. (2001) Segmental organization of neural crest migration. *Mech. Dev*. **105**, 37–45.
5. Bronner-Fraser, M. and Stern, C. (1991) Effects of mesodermal tissues on avian neural crest cell migration. *Dev. Biol.* **143**, 213–217.
6. Pettway, Z., Guillory, G., and Bronner-Fraser, M. (1990) Absence of neural crest cells from the region surrounding implanted notochords in situ. *Dev. Biol.* **142**, 335–345.
7. Erickson, C.A., Duong, T.D., and Tosney, K.W. (1992) Descriptive and experimental analysis of the dispersion of neural crest cells along the dorsolateral path and their entry into ectoderm in the chick embryo. *Dev. Biol.* **151**, 251–272.
8. Oakley, R.A., Lasky, C.J., Erickson, C.A., and Tosney, K.W. (1994) Glycoconjugates mark a transient barrier to neural crest migration in the chicken embryo. *Development* **120**, 103–114.
9. Debby-Brafman, A., Burstyn-Cohen, T., Klar, A., and Kalcheim, C. (1999) F-Spondin, expressed in somite regions avoided by neural crest cells, mediates inhibition of distinct somite domains to neural crest migration. *Neuron* **22**, 475–488.
10. Ranscht, B. and Bronner-Fraser, M. (1991) T-cadherin expression alternates with migrating neural crest cells in the trunk of the avian embryo. *Development* **111**, 15–22.
11. Eickholt, B.J., Mackenzie, S.L., Graham, A., Walsh, F.S., and Doherty, P. (1999) Evidence for collapsin-1 functioning in the control of neural crest migration in both trunk and hindbrain regions. *Development* **126**, 2181–2189.
12. Gammill, L.S., Gonzalez, C., Gu, C., and Bronner-Fraser, M. (2006) Guidance of trunk neural crest migration requires neuropilin 2/semaphorin 3F signaling. *Development* **133**, 99–106.
13. Krull, C.E., Lansford, R., Gale, N.W., Collazo, A., Marcelle, C., Yancopoulos, G.D., Fraser, S.E., and Bronner-Fraser, M. (1997) Interactions of Eph-related receptors and ligands confer rostrocaudal pattern to trunk neural crest migration. *Curr. Biol*. **7**, 571–580.
14. Wang, H.U. and Anderson, D.J. (1997) Eph family transmembrane ligands can mediate repulsive guidance of trunk neural crest migration and motor axon outgrowth. *Neuron* **18**, 383–396.
15. Landolt, R.M., Vaughan, L., Winterhalter, K.H., and Zimmermann, D.R. (1995) Versican is selectively expressed in embryonic tissues that act as barriers to neural crest cell migration and axon outgrowth. *Development* **121**, 2303–2312.
16. Pettway, Z., Domowicz, M., Schwartz, N.B., and Bronner-Fraser, M. (1996) Age-dependent inhibition of neural crest migration by the notochord correlates with alterations in the S103L chondroitin sulfate proteoglycan. *Exp. Cell Res*. **225**, 195–206.
17. Dutt, S., Kleber, M., Matasci, M., Sommer, L., and Zimmermann, D.R. (2006) Versican V0 and V1 guide migratory neural crest cells. *J. Biol. Chem*. **281**, 12123–12131.
18. Perris, R., Perissinotto, D., Pettway, Z., Bronner-Fraser, M., Morgelin, M., and Kimata, K. (1996) Inhibitory effects of PG-H/aggrecan and PG-M/versican on avian neural crest cell migration. *FASEB J.* **10**, 293–301.
19. Perissinotto, D., Iacopetti, P., Bellina, I., Doliana, R., Colombatti, A., Pettway, Z., Bronner-Fraser, M., Shinomura, T., Kimata, K., Morgelin, M., et al. (2000) Avian neural crest cell migration is diversely regulated by the two major hyaluronan-binding proteoglycans PG-M/versican and aggrecan. *Development* **127**, 2823–2842.
20. Schweigreiter, R., Walmsley, A.R., Niederost, B., Zimmermann, D.R., Oertle, T., Casademunt, E., Frentzel, S., Dechant, G., Mir, A., and Bandtlow, C.E. (2004) Versican V2 and the central inhibitory domain of Nogo-A inhibit neurite growth via p75NTR/NgR-independent pathways that converge at RhoA. *Mol. Cell. Neurosci.* **27**, 163–174.
21. Henkemeyer, M., Orioli, D., Henderson, J.T., Saxton, T.M., Roder, J., Pawson, T., and Klein, R. (1996) Nuk controls pathfinding of commissural axons in the mammalian central nervous system. *Cell* **86**, 35–46.
22. Orioli, D., Henkemeyer, M., Lemke, G., Klein, R., and Pawson, T. (1996) Sek4 and Nuk receptors cooperate in guidance of commissural axons and in palate formation. *EMBO J*. **15**, 6035–6049.
23. Wang, H.U., Chen, Z.F., and Anderson, D.J. (1998) Molecular distinction and angiogenic interaction between embryonic arteries and veins revealed by ephrin-B2 and its receptor Eph-B4. *Cell* **93**, 741–753.
24. Kawasaki, T., Bekku, Y., Suto, F., Kitsukawa, T., Taniguchi, M., Nagatsu, I., Nagatsu, T., Itoh, K., Yagi, T., and Fujisawa, H. (2002) Requirement of neuropilin 1-mediated Sema3A signals in patterning of the sympathetic nervous system. *Development* **129**, 671–680.

**This article should be cited as follows:**

Dutt, S., Matasci, M., Sommer, L., and Zimmermann, D.R. (2006) Guidance of neural crest cell migration: the inhibitory function of the chondroitin sulfate proteoglycan, versican. *TheScientificWorldJOURNAL* 6, 1114–1117. DOI 10.1100/tsw.2006.219.
